# Correlation between Activation of PI3K/AKT/mTOR Pathway and Prognosis of Breast Cancer in Chinese Women

**DOI:** 10.1371/journal.pone.0120511

**Published:** 2015-03-27

**Authors:** Ling Deng, Jie Chen, Xiao Rong Zhong, Ting Luo, Yan Ping Wang, Hui Fen Huang, Li-Juan Yin, Yan Qiu, Hong Bu, Qing Lv, Hong Zheng

**Affiliations:** 1 Cancer Center, West China Hospital, Sichuan University, Chengdu, Sichuan Province, China; 2 Laboratory of Molecular Diagnosis of Cancer, State Key Laboratory of Biotherapy, National Collaborative Innovation Center for Biotherapy, West China Hospital, Sichuan University, Chengdu, Sichuan Province, China; 3 Department of Thyroid and Breast Surgery, West China Hospital, Sichuan University, Chengdu, Sichuan Province, China; 4 Department of Pathology, West China Hospital, Sichuan University, Chengdu, Sichuan Province, China; IPATIMUP/Faculty of Medicine of the University of Porto, PORTUGAL

## Abstract

**Background:**

Abnormal activation of PI3K/AKT/mTOR (PAM) pathway, caused by *PIK3CA* mutation, *KRAS* mutation, PTEN loss, or *AKT1* mutation, is one of the most frequent signaling abnormalities in breast carcinoma. However, distribution and frequencies of mutations in PAM pathway are unclear in breast cancer patients from the mainland of China and the correlation between these mutations and breast cancer outcome remains to be identified.

**Methods:**

A total of 288 patients with invasive ductal breast cancer were recruited in this study. Mutations in *PIK3CA* (exons 4, 9 and 20), *KRAS* (exon 2) and *AKT1* (exon 3) were detected using Sanger sequencing. PTEN loss was measured by immunohistochemistry assay. Correlations between these genetic aberrations and clinicopathological features were analyzed.

**Results:**

The frequencies of *PIK3CA* mutation, *KRAS* mutation, *AKT1* mutation and PTEN loss were 15.6%, 1.8%, 4.4% and 35.3%, respectively. However, except for PTEN loss, which was tied to estrogen receptor (ER) status, these alterations were not associated with other clinicopathological features. Survival analysis demonstrated that *PIK3CA* mutation, PTEN loss and PAM pathway activation were not associated with disease-free survival (DFS). Subgroup analysis of patients with ER positive tumors revealed that *PIK3CA* mutation more strongly reduced DFS compared to wild-type *PIK3CA* (76.2% vs. 54.2%; ***P* = 0.011**). *PIK3CA* mutation was also an independent factor for bad prognosis in ER positive patients.

**Conclusions:**

*AKT1*, *KRAS* and *PIK3CA* mutations and PTEN loss all exist in women with breast cancer in the mainland China. *PIK3CA* mutation may contribute to the poor outcome of ER positive breast carcinomas, providing evidence for the combination of PI3K/AKT/mTOR inhibitors and endocrine therapy.

## Introduction

The PI3K/AKT/mTOR (PAM) pathway is central to the control of cell transcription, translation, migration, metabolism, proliferation and survival [1]. Epidemiological and preclinical studies have confirmed that PAM pathway plays an important role in the progression of human tumors [2], and that it is a key factor to regulate tumor angiogenesis and tumor cell metabolism. Abnormal activation of PAM pathway is one of the most common tumor-related signaling abnormalities that can be detected in a variety of tumors including breast cancer, colorectal cancer, endometrial carcinoma, lung cancer and glioblastoma [1,3–5]. This abnormal activation involves multiple molecular alterations, mainly including *PIK3CA* mutation, PTEN loss and *AKT1* mutation. In addition, *KRAS* mutation and *BRAF* mutation from the MAPK pathway can also result in PAM activation [6–8].

Mutation in *PIK3CA*, a phosphatidylinositol 3-kinase (PI3K) subunit encoding p110α, is the most frequent type of *PI3K* alteration. It occurs with a mutation frequency of 18–40% in breast cancer, and has mutational hot spots at E542K, E545K (exon 9), and H1047R (exon20) [9–11]. *PIK3CA* mutation enhances the activity of PI3K lipase and thus upregulates the downstream AKT activity. As a negative feedback regulating factor of PAM pathway, PTEN is absent in 25% and mutated in less than 5% breast cancer patients [12]. And also, PTEN inactivation correlates with increased phosphorylation of AKT, mTOR and S6K1. Mutation of *AKT1*, a downstream molecule of PI3K, occurs in 5–24% breast cancers [13]. This mutation activates AKT1 by means of PI3K-independent localization to the plasma membrane and stimulates downstream mTOR signaling [14]. Approximately 5% of breast cancer patients harbor *KRAS* mutations in the MAPK pathway, leading to continuous activation of PI3K [15], and the frequency of *KRAS* mutation is 1.56% in the COSMIC database (http://cancer.sanger.ac.uk/cancergenome/projects/cosmic/). All of these alterations result in PAM pathway activation, which is vital for tumor development.

A preclinical study has demonstrated that PAM pathway inhibition has anti-proliferative activity in a variety of breast cancer cell lines, including HER2 over-expressing cells resistant to trastuzumab and lapatinib [16]. Currently, several agents targeted at PAM pathway have entered phase I, II or III clinical trials, including PI3K inhibitor, mTOR inhibitor, PI3K/mTOR inhibitor and AKT inhibitor. Systematic determination of molecular changes in this pathway is sure to help us understand the exact mechanism of these inhibitors and guide their clinical application. Reports on the distribution and frequency of mutations in PAM pathway are still rare in breast cancer patients from the Chinese mainland and the correlation between mutations in the pathway and outcome of breast cancer remains to be identified. Therefore, we conducted this study to determine the frequencies of *PIK3CA*, *AKT* and *KRAS* mutations and PTEN loss, and analyze the relationship of the mutations with clinicopathological features and prognosis.

## Materials and Methods

### Patients and specimens

More than 9,000 patients were registered in the Breast Cancer Information Management System of West China Hospital, Sichuan University. In this study, patients with ductal breast cancer were recruited from 1084 patients, who had undergone surgery in the Department of Thyroid and Breast Surgery of West China Hospital, Sichuan University from January 2005 to May 2008. Patients (a total of 796 cases) who failed follow-up, underwent neoadjuvant chemotherapy, lacked complete clinical information or were unable to provide a sufficient amount of tumor tissue sample were excluded from the study. Thus, 288 patients were finally included. Histological diagnosis of breast cancer was confirmed by experienced pathologists at the Department of Pathology, West China Hospital, Sichuan University. Formalin fixed paraffin-embedded tumor tissues were prepared from each patient and tumor content of over 80% were confirmed. A total of 120μm of tissue sections from each case were used for DNA extraction and sequencing to detect mutations in *PIK3CA* (exons 4, 9, 20), *KRAS* (exon 2) and *AKT1* (exon 3). Five tissue slides, 4μm for each, were prepared for PTEN loss detection. The study was approved by the independent Clinical Trials and Biomedical Ethics Special Committees, West China Hospital, Sichuan University, and written informed consent of the patients was obtained. Comprehensive postoperative treatments (chemotherapy, radiation therapy, endocrine therapy and targeted therapy) were administered according to NCCN guidelines. Clinicopathological features are summarized in Table 1. Follow-up (physical examination, blood tests, X-ray mammography, CT scan of head, chest and abdomen, and bone scintigraphy) was performed every three months within the first 2 years after surgery, and then every 6 months. The cut-off of follow-up was May 2013.

**Table 1:** Clinicopathological features of the 288 breast cancer patients.

Items	Number of patients (%)
Menopause	
Yes	135 (46.9%)
No	153 (53.1%)
Tumor size (cm)	
T1 (T≤2)	79 (27.4%)
T2 (2<T≤5)	167 (58.0%)
T3 or larger (T>5)	42 (14.6%)
Lymph node involvement	
Positive	160 (55.6%)
Negative	128 (44.4%)
Tumor grade	
1	7 (2.4%)
2	84 (29.2%)
3	197 (68.4%)
ER status	
Positive	173 (60.0%)
Negative	115 (40.0%)
PR status	
Positive	192 (66.7%)
Negative	96 (33.3%)
HER2 status	
Positive	24 (8.3%)
Negative	261 (90.6%)
Unknown	3 (1.1%)
Ki67 index	
<14%	83 (28.8%)
≥14%	205 (71.2%)

### Analysis of *PIK3CA*, *AKT1* and *KRAS* mutations

DNA extraction from the paraffin-embedded tissue sections was performed using TIANamp FFPE DNA Kit (TIANGEN BIOTECH, Beijing, China), according to the manufacturer's instruction. PCR was performed for amplification of *PIK3CA* exons 4, 9, 20, *AKT1* exon 3 and *KRAS* exon 2. The primers used are listed in Table 2. PCR products were sequenced using an ABI 3730XL sequencer. The sequencing peak chromatogram files were imported into SMD software (Sequence Mutation Detector, CapitalBio Corporation) and compared with the reference sequences of *PIK3CA* (accession no.NC_000003.11), *AKT1* (accession no.NC_000014.8) and *KRAS* (accession no.NC_000012.11) in Genbank to determine mutations.

**Table 2:** The primers of *AKT1* exon 3, *KRAS* exon2 and *PIK3CA* exons 4, 9 and 20.

Items	Primers
*KRAS* exon 2	Forward: ACTGGTGGAGTATTTGATAGTGTAT
	Reverse: TATCTGTATCAAAGAATGGTCCT
*AKT1* exon 3	Forward: CTGGCGAGGGTCTGACGGGT
	Reverse: CAGTGCTTGTTGCTTGCCAG
*PIK3CA* exon 4	Forward: TAAAATGAAAAACCTTACAGGAAAT
	Reverse: AGTGCAAGAAAAAGGTTATCTAAAA
*PIK3CA* exon 9	Forward: CAGTTAATTAGCAATGTAAAA
	Reverse: ATTCTGCTTTATTTATTCC
*PIK3CA* exon 20	Forward: GCAAAGACCTGAAGGTATTAAC
	Reverse: GTGGAATCCAGAGTGAGCTT

### Immunohistochemistry of PTEN expression

Immunohistochemistry (IHC) for PTEN expression was performed as previously described [17]. After deparaffinization and rehydration, the tissue slides were incubated away from light in 3% H_2_O_2_ solution for 15 minutes. A 40-min incubation at 98°C with Target Retrieval Solution pH 9 (EDTA, pH 9, Gene Tech, Shanghai, China) was performed in a water bath. The slides were then cooled to room temperature (for at least 30 min). Next, a 4°C overnight incubation with monoclonal mouse anti-human PTEN antibody (dilution 1:100, clone 6H2.1, Dako) was performed, followed by a 30-min incubation with peroxidase-labeled polymer conjugated to goat anti-mouse immunoglobulins (EnVision/HRP, Dako). The final step was a chromogenic reaction using 3, 3’-diaminobenzidine chromogen solution. The IHC assay for each sample was repeated 5 times. Normal breast tissues, obtained from Cancer Molecular Diagnostics Laboratory of West China Hospital, Sichuan University, served as positive controls, and homotype mouse anti-human IgGa (BioLegend) as negative control. Senior pathologists were responsible for interpretation and scoring of the IHC results. The scoring system was described previously [17]. Normal surrounding epithelium served as internal control. Tumor tissues were scored as the following: score 2 = same staining intensity as that of normal epithelium; score 1 = staining intensity weaker than normal; score 0 = no staining. We defined scores 0 and 1 as loss of PTEN.

### Statistical analysis

Chi-square (χ^2^) test and Fisher’s exact test were performed to assess significance of the association between variables (*PIK3CA*, *AKT*, *KRAS* mutations and PTEN loss in breast cancer tissues) and clinical characteristics. Kaplan-Meier survival curve was drawn and tested by Log-rank test to evaluate the differences in disease-free survival between the variables. The effect of mutations on prognosis was analyzed by univariate and COX multivariate risk models. Statistical difference was defined as *P*<0.05.

## Results

### Clinicopathological characteristics

The clinicopathological characteristics of the 288 cases of sporadic breast cancer were listed in Table 1. Median follow up duration was 66 months (range, 3–87 months). Out of the 288 patients, 84 suffered relapse, including: 11 with ipsilateral local recurrence, 5 with contralateral recurrence, 19 with pulmonary metastasis, 17 with liver metastasis, 19 with bone metastasis, 7 with brain metastasis, 5 with supraclavicular lymph node involvement, and 1 with laryngeal involvement.

### *PIK3CA*, *AKT* and *KRAS* mutations and PTEN loss in 288 sporadic breast cancer patients

**Fig 1:**
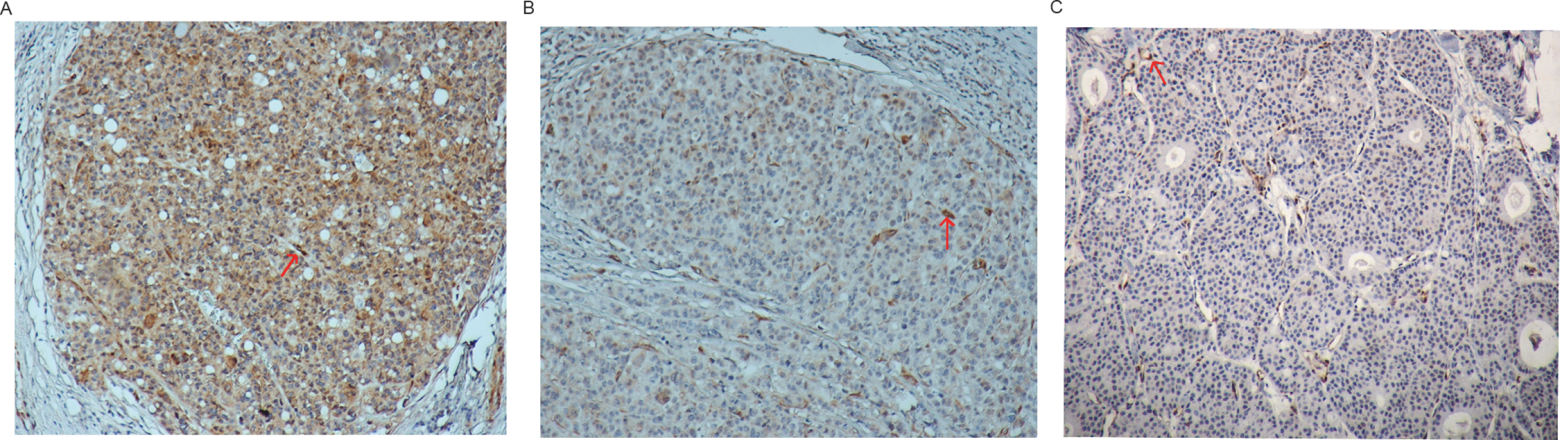
Immunohistochemistry of PTEN expression. Depicted are photomicrographs of PTEN scoring: A, score 2 = same staining intensity as of surrounding normal epithelium; B, score 1 = weaker than normal; C, score 0 = no staining (× 100). The red arrow indicated the normal tissue.

*PIK3CA*, *AKT*, *KRAS* mutation and PTEN loss rates are shown in Table 3. One specimen carried *PIK3CA* gene double mutations in exon 9 and exon 20. The IHC scoring based on PTEN expression assessed for 278 patients showed 51 cases of score 0, 47 cases of score 1, and 180 cases of score 2 (Fig. 1). The other 10 cases fell into failure of staining or nonassessable results. PTEN loss (scores 0 and 1) occurred in 35.3% of sporadic breast cancer patients, including 4 who presented with both PTEN loss and *PIK3CA* mutation. By defining that mutation in *PIK3CA*, *AKT1* or *KRAS*, or loss of PTEN expression will lead to PAM pathway activation, 146 of the 288 patients were discovered to have PAM activation in their tumors.

**Table 3:** Alterations of genes in 288 breast invasive ductal carcinomas.

	Positive rate	Yes	No	Failure
***KRAS mutation***	1.8% (5/278)	5	273	10
***AKT1* mutation**	4.4% (11/252)	11	241	36
***PIK3CA* mutation**	15.6% (39/250)	39†	211§	38¶
***PIK3CA* exon 4 mutation**	1.4% (3/216)	3	213	72
***PIK3CA* exon 9 mutation**	4.7% (10/214)	10	204	74
***PIK3CA* exon 20 mutation**	12.3% (27/219)	27	192	69
PTEN loss*	35.3% (98/278)	98	180	10
**PAM activation**	50.7% (146/288)	146‡	142	-

†, *PIK3CA* mutation with any exon

§, no mutation in any exon

¶, Amplification failure of 3 exons

*, PTEN loss by immunohistochemistry

‡, Alteration in any molecule of *PIK3CA*, *AKT1*, *KRAS* or PTEN.

The correlation between clinicopathological characteristics and overall PAM activation, *PIK3CA* mutation and PTEN loss is presented in Table 4. PAM pathway activation and *PIK3CA* mutation did not seem to correlate with clinicopathological features (menopause, histological grade, ER status, PR status, HER2 status, Ki67 expression, tumor size and lymph node involvement). PTEN loss was tied to ER status, but not associated with other clinicopathological features. Correlation analysis of the presence of *AKT1* and *KRAS* mutations was not performed due to the extremely low frequency found in our work.

**Table 4:** Correlation between clinicopathological features and PAM pathway activation, *PIK3CA* mutation and PTEN loss.

	PAM activation		*PIK3CA* mutation		PTEN loss	
Items	(146 cases)		(39 cases)		(98 cases)	
	N (%)	*P*	N (%)	*P*	N (%)	*P*
Menopause						
Yes	63(43.2%)	0.238	16(41.0%)	0.6	47(48.0%)	0.151
No	83(56.8%)		23(59.0%)		51(52.0%)	
Tumor size (cm)						
T1 (T≤2)	34(23.3%)	0.246	7(17.9%)	0.345	26(26.5%)	0.772
T2 (2<T≤5)	88(60.3%)		25(64.2%)		59(60.2%)	
T3 or larger (T>5)	24(16.4%)		7(17.9%)		13(13.3%)	
Lymph node involvement						
Positive	82(56.2%)	0.906	23(59.0%)	0.487	51(52.0%)	0.376
Negative	64(43.8%)		16(41.0%)		47(48.0%)	
Tumor grade						
1	4(2.7%)	0.711	2(5.1%)	0.421	1(1.0%)	0.427
2	45(30.8%)		9(23.1%)		31(31.7%)	
3	97(66.5%)		28(71.8%)		66(67.3%)	
ER status						
Positive	83(56.8%)	0.28	24(61.5%)	0.918	52(53.1%)	***0*.*041***
Negative	63(43.2%)		15(38.5%)		46(46.9%)	
PR status						
Positive	93(63.7%)	0.318	22(56.4%)	0.138	62(63.3%)	0.284
Negative	53(36.3%)		17(43.6%)		36(36.7%)	
HER2 status						
Positive	13(8.9%)	0.833	5(12.8%)	0.359	8(8.2%)	0.94
Negative	133(91.1%)		34(87.2%)		90(91.8%)	
Unknown	0		0		0	
Ki67 index						
≥14%	107(73.3%)	0.438	32(82.1%)	0.174	74(75.5%)	0.269
<14%	39(26.7%)		7(17.9%)		24(24.5%)	

### Effect of PAM activation, *PIK3CA* mutation and PTEN loss on prognosis

**Fig 2:**
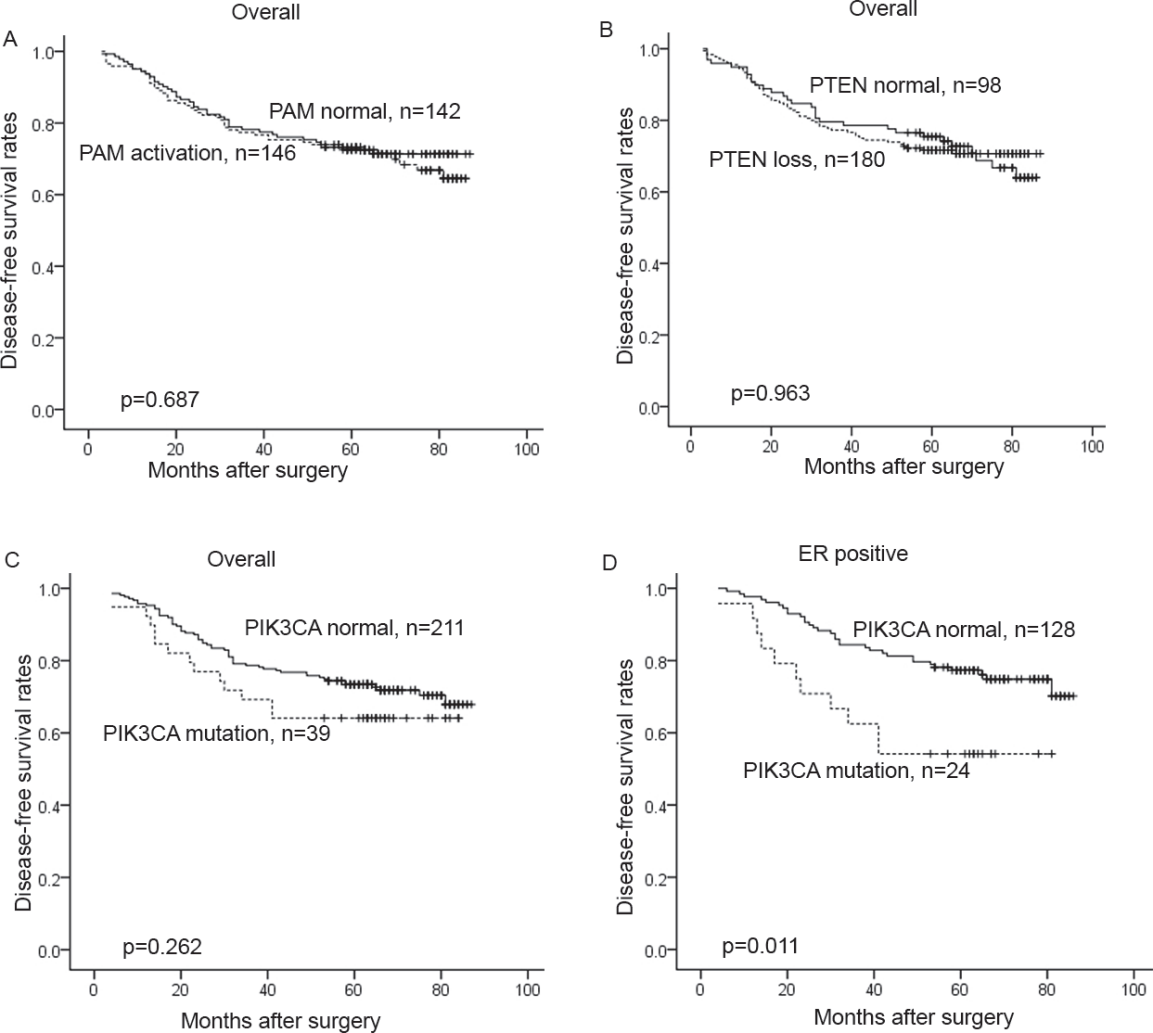
Correlation between PI3K/AKT/mTOR pathway alterations and prognosis by Kaplan–Meier survival analysis. A. PAM activation vs. normal PAM in all patients; B. PTEN loss vs. normal PTEN in all patients; C. mutant *PIK3CA* vs. normal *PIK3CA* in all patients; D. mutant *PIK3CA* vs. normal *PIK3CA* in ER positive patients.

The 5 year disease-free survival (DFS) rate was 71.4% in PAM pathway-inactivated patients and 72.4% in those with PAM activation. The difference was not statistically significant by Kaplan-Meier survival analysis and Log-rank test, *P* = 0.687 (HR = 1.091; 95%CI, 0.712–1.671; Fig. 2A). Among the 278 patients who successfully underwent detection of PTEN loss, 5-year DFS of patients without PTEN loss was not significantly different from that of those with PTEN loss (74.2% vs. 70.7%; *P* = 0.963; HR = 1.011; 95%CI, 0.644–1.585; Fig. 2B). *PIK3CA* mutation status was successfully analyzed in 250 patients. Among these, 5-year DFS rate was 72.6% in patients without mutation and 64.1% in patients with mutation. The difference was not statistically significant (*P* = 0.262; HR = 1.392; 95%CI, 0.778–2.491; Fig. 2C). Subsequently we conducted subgroup analysis in 152 ER positive breast cancers and found that there was a statistically significant difference in 5-year DFS between ER positive *PIK3CA* mutant and *PIK3CA* wild-type patients (54.2% vs. 76.2%; ***P* = 0.011**; HR = 2.383; 95%CI, 1.199–4.738; Fig. 2D). Univariate and multivariate analysis of 152 ER positive breast cancer patients (the baseline was summarized in Table 5) revealed that *PIK3CA* mutation was an independent predictive factor for worse prognosis (Table 6).

**Table 5:** Correlation between *PIK3CA* mutation and clinicopathologic characteristics of 152 ER-positive sporadic breast cancer patients.

		PIK3CA mutation	
	Mutant (n = 24)	Wild (n = 128)	*P* value
Menopause			
Yes	8(33.3%)	64(50%)	0.133
No	16(66.7%)	64(50%)	
Tumor grade			
1/2	8(33.3%)	57(44.5%)	0.309
3	16(66.7%)	71(55.5%)	
PR status			
Negative	9(37.5%)	16(12.5%)	0.002
Positive	15(62.5%)	112(87.5%)	
Her-2 status			
Negative	22(91.7%)	120(93.8%)	0.592
Positive	2(8.3%)	7(5.5%)	
Unknown	0	1(0.7%)	
Ki67 index			
<14%	6(25%)	51(39.8%)	0.168
≥14%	18(75%)	77(60.2%)	
Tumor size (cm)			
T1 (T≤2)	3(12.5%)	38(29.7%)	0.217
T2 (2<T≤5)	16(66.7%)	70(54.7%)	
T3 or larger (T>5)	5(20.8%)	20(15.6%)	
Lymph node involvement			
Negative	8(33.3%)	55(43.0%)	0.379
Positive	16(66.7%)	73(57.0%)	

**Table 6:** PIK3CA mutation in 152 ER positive breast cancers.

Items	Univariate		Multivariate	
	HR * (95% CI)	*P* value	HR* (95% CI)	***P* value**
Menopause	0.813(0.447–1.479)	0.498	0.857(0.439–1.671)	0.65
Tumor size	2.606(1.357–5.006)	***0*.*004***	2.151(1.067–4.337)	***0*.*032***
Lymph node involvement	2.034(1.044–3.961)	***0*.*037***	1.688(0.844–3.375)	0.139
Tumor grade	1.111(0.605–2.041)	0.734	0.857(0.439–1.671)	0.65
PR	2.817(1.467–5.410)	***0*.*002***	2.102(0.388–1.376)	***0*.*029***
HER2	0.670(0.162–2.771)	0.58	0.747(0.172–3.251)	0.698
Ki67	1.230(0.656–2.309)	0.519	1.530(0.769–3.042)	0.225
*PIK3CA*	2.383(1.199–4.738)	***0*.*013***	2.102(1.016–4.349)	***0*.*045***

Abbreviation: HR, Hazard ratio; CI, confidence interval.

*, Hazard ratio of premenopausal against postmenopausal; tumor size >5 against tumor size ≤5; lymph node positive against negative; tumor grade 3 against grade 1/2; PR-negative against PR-positive; HER2 positive against negative; Ki67≥14% against Ki67<14%; PIK3CA mutation against wild type.

### Mutation frequencies in relapsed patients and relapse-free patients

We further conducted subgroup analysis and compared the mutation frequencies in 48 patients who relapsed within 3 years after surgery and 75 patients who were relapse-free over 6 years. Table 7 presents basic clinicopathological information of the two groups. The relapsed patients appeared to have larger tumor size and higher positive hormone receptor rate, but they were not significantly different from relapse-free patients in other clinicopathological features. Mutation rates of *PIK3CA* and *PIK3CA/AKT/KRAS* were statistically higher in the relapsed subgroup than that in the relapse-free subgroup, but there was no significant difference in PTEN loss between the two subgroups. Further analysis of the 48 relapsed patients revealed that *PIK3CA* and *PIK3CA/AKT/KRAS* mutations were more frequent in hormone receptor positive or ER positive cases (Table 8).

**Table 7:** Clinicopathological features of 48 relapsed patients vs. 75 relapse-free patients.

Items	Number of patients		
	48 cases	75 cases	*P* value
Menopause			
Yes	23 (47.9%)	33 (44%)	0.713
No	25 (52.1%)	42 (56%)	
Tumor size (cm)			
T1 (T≤2)	12 (25%)	29 (38.7%)	***0*.*004***
T2 (2<T≤5)	20 (41.7%)	39 (52.0%)	
T3 or lager (T>5)	16 (33.3%)	7 (9.3%)	
Lymph node involvement			
N0	15 (31.3%)	35 (46.7%)	0.052
N1	10 (20.8%)	23 (30.7%)	
N2	23 (47.9%)	17 (22.7%)	
Tumor grade			
2	14 (29.2%)	23 (30.7%)	0.86
3	34 (70.8%)	52 (69.3%)	
Hormone receptor status			
Positive	38 (79.2%)	45 (60%)	***0*.*031***
Negative	10 (20.8%)	30 (40%)	
HER2 status			
Positive	3 (6.3%)	6 (8.0%)	0.745
Negative	45 (93.7%)	69 (92.0%)	
Ki67 index			
≥14%	32 (66.7%)	50 (66.7%)	1
<14%	16 (33.3%)	25 (33.3%)	
*PIK3CA* †			
Mutation	11 (25%)	4 (6.3%)	***0*.*006***
Wild type	33 (75%)	59 (93.7%)	
PIK3CA/AKT/KRAS*			
Mutation	16 (34.0%)	7 (9.6%)	***0*.*001***
Wild type	31 (66.0%)	66 (90.4%)	
PTEN loss¶			
Yes	16 (34.0%)	29 (40.3%)	0.493
No	31 (66.0%)	43 (59.7%)	
PAM activation			
Yes	30 (62.5%)	35 (46.7%)	0.086
No	18 (37.5%)	40 (53.3%)	

†, successful analysis of *PIK3CA* sequencing, 44 cases vs. 63 cases

*, Mutation in any gene of *PIK3CA*, *AKT*, *KRAS*, 47 cases vs. 73 cases

¶, successful analysis of PTEN IHC, 47 cases vs. 72 cases.

**Table 8:** Correlation between *PIK3CA*, *KRAS/PIK3CA/AKT* mutation and hormone receptor status of 48 relapsed patients.

	*PIK3CA* mutant		*KRAS/PIK3CA/AKT* mutant	
	n = 11	P*	n = 16	P*
ER and/or PR status				
Positive	11 (100%)	***0*.*046***	16 (100%)	***0*.*010***
Negative	0		0	
ER status				
Positive	10 (90.9%)	0.076	15 (93.8%)	***0*.*008***
Negative	1 (9.1%)		1 (6.2%)	
PR status				
Positive	6 (54.5%)	1.000	9 (56.3%)	1.000
Negative	5 (45.5%)		7 (43.7%)	

* Fisher’s exact test

## Discussion

PAM pathway is central to the control of cell growth, migration and survival. Given the fact that abnormal activation of PAM pathway closely correlates with the efficacy of medical treatment for breast carcinoma including endocrine therapy, anti-HER2 therapy and chemotherapy, research in this area is clinically important. In the present study, the mutation rate of *PIK3CA* in breast carcinoma was 15.6% (39/250), lower than that reported in white [9,18] and Japanese populations [19]. In agreement with literature, our study also found that *PIK3CA* mutations occurred at hot spots E545K and H1047R. The mutation rate of *KRAS* and *AKT* in our study was 1.8% and 4.4%, respectively, again consistent with that in the literature. However, in our patients, the mutation rate of *PIK3CA* in the ER positive breast cancer patients was found to be only 15.8% (24/152), which is significantly lower than that of 28–47% in the literature [9,13,20,21]. This observation might be potentially attributed to ethic differences.

PAM pathway plays an important role in resistance to anti-HER2 therapy in HER2-positive breast cancer. In early or advanced HER2-positive breast cancer, *PIK3CA* mutation or PAM pathway activation is associated with poor prognosis [22–24]. We chose 33 cases of HER-2 positive breast cancer frozen tissue (18 cases with Lumina B like, 15 cases with HER2-positive type) for sequencing of 58 exons of the *mTOR gene* (next generation sequencing, Ion Torrent), nevertheless mutations that could cause an amino acid change were not found (data not shown). Indeed, mTOR is a key downstream molecule of the PAM pathway, and the mutation rate of the *mTOR* gene is extremely rare in the reported data. In the COSMIC database, *mTOR* mutation was found in 20 breast cancers; moreover, the frequency of *mTOR* mutation was only 2.45% in all types of tumors that have been reported. Therefore, a larger number of samples with statistical significance are required in order to determine the role of *mTOR* mutation in PAM pathway activation.

Loss of PTEN can also activate the PAM pathway. The occurrence of PTEN loss is approximately 25% in breast cancers. In ER positive tumors, it reaches 37–44% [20,25,26]. The relationship of this mutation to clinicopathological markers and prognosis remains unknown, but it was found to have a correlation with lymph node involvement, loss of ER staining, higher tumor grade, TNM staging and disease-related death [27,28]. Loss of PTEN was associated with shorter relapse-free survival in Tamoxifen-treated ER positive patients [26]; however, most of the research did not reveal an association between PTEN loss and prognosis. In addition, Iqbal *et al* reported that loss of PTEN protein predicted early recurrence in triple-negative breast cancer [29]. In the present study, the rate of PTEN loss was 35.3%, and PTEN loss was associated with ER status. We did not find an association between PTEN loss and prognosis in 54 triple-negative breast cancers (data not shown).

It was found that *PIK3CA* mutation was associated with ER positive, small size, negative HER2 status [20], but correlation between this mutation and patient prognosis is still to be determined. Actually, the effect of *PIK3CA* mutation on the prognosis of breast cancer should be assessed according to different cancer molecular subtypes. The relationship between *PIK3CA* mutation and outcome of ER positive breast cancer remains unclear. It had been reported that *PIK3CA* mutation was associated with better outcome in ER positive breast cancer [19–21]. But in other reports this association did not exist [13]. Li *et al* found that patients with *PIK3CA* mutation had a poorer outcome in ER positive breast cancer [30]. Studies have found that exon 20, encoding the kinase domain, was tied to the prognosis of patients; and exon 9, coding helical domains, was not associated with patients’ outcome [21]. When exon 9 and exon 20 were analyzed together, *PIK3CA* mutation was not associated with prognosis in ER positive breast cancer patients [31]. But subgroup analysis revealed that *PIK3CA* mutants had a similar survival compared to the good-prognosis, low-proliferative subgroup (*P*>0.05) and a better outcome compared with the poor-prognosis, highly proliferative subtype (*P*<0.05) [31]. The present study indicated that ER positive breast cancer patients with *PIK3CA* mutation had poorer outcome. Univariate and multivariate analysis confirmed that this mutation is an independent adverse prognostic factor in ER positive breast carcinoma (57 cases with Lumina A like, 95 cases with Lumina B like). These results suggest that *PIK3CA* mutation may contribute to poor outcome or failure of medical treatment in ER positive early breast cancer patients.

Subsequently, we further analyzed our results between the 48 patients with poorer prognosis (relapsed within 3 years after surgery) and the 75 patients with better outcome (over 6 years of relapse-free). It was found that mutation rates of *PIK3CA* and *KRAS/PIK3CA/AKT* in recurrent patients were significantly higher than those in relapse-free patients. In addition, *PIK3CA* and *KRAS/PIK3CA/AKT* mutations appeared to occur more frequently in hormone receptor or ER positive relapsed patients. All the results further validated that activation of PAM pathway correlates with poor outcome in ER positive cancers, indicating that ER positive breast cancer patients harboring these mutations are likely to have a poorer prognosis. In clinical practice, about 40% of patients failed to respond to or were resistant to routine endocrine therapy [32]. Resistance to endocrine therapy is a long process of accumulation, which might be affected by many factors. Increasing attention is paid to the connection between endocrine therapy resistance and the increased expression or signaling overaction of growth factor receptor pathways, including PAM [33]. Both preclinical and clinical studies indicated that resistance to endocrine therapy might be reversed by PAM pathway inhibitors. mTORC1 activates ER by a non-hormone-dependent mode [34]. Estradiol inhibits apoptosis by blocking PI3K/mTOR [35]. Endocrine-resistant breast cancer cells were found to carry high PAM pathway activation [36]. These studies revealed the interaction between ER and the PAM pathway, and this might explain why *PIK3CA* mutation only affected the ER positive patient population, but not the ER negative patients. *In vitro*, everolimus combined with tamoxifen, fulvestrant, letrozole in ER positive breast cancer cells showed a synergistic effect [37]. The results of BOLERO-2 showed that in hormone receptor positive advanced breast cancer, everolimus in combination with exemestane significantly improved PFS [38]. The present study confirmed that the PAM pathway is excessively activated in relapsed hormone receptor or ER positive breast cancer patients, suggesting that PAM pathway activation may contribute to the failure of endocrine treatment and that PAM pathway inhibitors may reverse endocrine therapy resistance, providing evidence for the combination of PAM pathway inhibitors and hormonal therapy.

Based on survival analysis of the *PIK3CA* mutation, *AKT1* mutation, *KRAS* mutation and PTEN loss in the 152 ER positive breast cancer patients, we found that only *PIK3CA* mutation was a predictive factor. Furthermore, PAM activation in ER positive patients was not shown to be a predictive factor. In addition, in the 98 ER negative breast cancer patients, none of *PIK3CA* mutation, *AKT1* mutation, *KRAS* mutation and PTEN loss were associated with the patients’ prognosis by Kaplan-Meier curve and COX analysis (*P*>0.05).

Several caveats should be taken into account for the present study. First, it was a single-center retrospective study. Further evaluation of the PAM pathway and its inhibitors in clinical use requires a large prospective controlled study with long-term follow up. Second, mutations at other loci and their effects on prognosis are still to be studied. Third, we did not use paired normal tissue or blood samples as controls in this study, we relied upon the background information from previous studies to ensure that the mutations found were real, because as this was a retrospective study, normal control tissues had not been stored and were therefore unavailable. In addition, the relationship between mutations and molecular subtype of breast cancer and the internal molecular mechanism remains to be further elucidated.

In conclusion, this is the first study to investigate PAM pathway alterations in sporadic breast cancer in China. Results of the study preliminarily showed that *PIK3CA* mutation predicts outcome of ER positive tumors, providing biomolecular evidence for the combination use of PI3K inhibitors and endocrine therapy.

## Supporting Information

S1 Dataset (XLS)

## References

[1] Liu P, Cheng H, Roberts TM, Zhao JJ. Targeting the phosphoinositide 3-kinase pathway in cancer. Nat Rev Drug Discov. 2009; 8: 627–644. 10.1038/nrd2926 19644473 PMC3142564

[2] Wymann MP, Zvelebil M, Laffargue M. Phosphoinositide 3-kinase signalling—which way to target? Trends Pharmacol Sci. 2003; 24: 366–376. 12871670 10.1016/S0165-6147(03)00163-9

[3] Kita D, Yonekawa Y, Weller M, Ohgaki H. PIK3CA alterations in primary (de novo) and secondary glioblastomas. Acta Neuropathol. 2007; 113: 295–302. 17235514 10.1007/s00401-006-0186-1

[4] Wu G, Xing M, Mambo E, Huang X, Liu J, Guo Z, et al Somatic mutation and gain of copy number of PIK3CA in human breast cancer. Breast Cancer Res. 2005; 7: R609–616. 16168105 10.1186/bcr1262 PMC1242128

[5] Kawano O, Sasaki H, Okuda K, Yukiue H, Yokoyama T, Yano M, et al PIK3CA gene amplification in Japanese non-small cell lung cancer. Lung Cancer. 2007; 58: 159–160. 17681398 10.1016/j.lungcan.2007.06.020

[6] Markman B, Atzori F, Perez-Garcia J, Tabernero J, Baselga J. Status of PI3K inhibition and biomarker development in cancer therapeutics. Ann Oncol. 2010; 21: 683–691. 10.1093/annonc/mdp347 19713247

[7] Populo H, Soares P, Faustino A, Rocha AS, Silva P, Azevedo F, et al mTOR pathway activation in cutaneous melanoma is associated with poorer prognosis characteristics. Pigment Cell Melanoma Res. 2011; 24: 254–257. 10.1111/j.1755-148X.2010.00796.x 21029395

[8] Faustino A, Couto JP, Populo H, Rocha AS, Pardal F, Cameselle-Teijeiro JM, et al mTOR pathway overactivation in BRAF mutated papillary thyroid carcinoma. J Clin Endocrinol Metab. 2012; 97: E1139–1149. 10.1210/jc.2011-2748 22549934

[9] Campbell IG, Russell SE, Choong DY, Montgomery KG, Ciavarella ML, Hooi CS, et al Mutation of the PIK3CA gene in ovarian and breast cancer. Cancer Res. 2004; 64: 7678–7681. 15520168 10.1158/0008-5472.CAN-04-2933

[10] Karakas B, Bachman KE, Park BH. Mutation of the PIK3CA oncogene in human cancers. Br J Cancer. 2006; 94: 455–459. 16449998 10.1038/sj.bjc.6602970 PMC2361173

[11] Samuels Y, Wang Z, Bardelli A, Silliman N, Ptak J, Szabo S, et al High frequency of mutations of the PIK3CA gene in human cancers. Science. 2004; 304: 554 15016963 10.1126/science.1096502

[12] Hennessy BT, Smith DL, Ram PT, Lu Y, Mills GB. Exploiting the PI3K/AKT pathway for cancer drug discovery. Nat Rev Drug Discov. 2005; 4: 988–1004. 16341064 10.1038/nrd1902

[13] Stemke-Hale K, Gonzalez-Angulo AM, Lluch A, Neve RM, Kuo WL, Davies M, et al An integrative genomic and proteomic analysis of PIK3CA, PTEN, and AKT mutations in breast cancer. Cancer Res. 2008; 68: 6084–6091. 10.1158/0008-5472.CAN-07-6854 18676830 PMC2680495

[14] Carpten JD, Faber AL, Horn C, Donoho GP, Briggs SL, Robbins CM, et al A transforming mutation in the pleckstrin homology domain of AKT1 in cancer. Nature. 2007; 448: 439–444. 17611497 10.1038/nature05933

[15] Janku F, Lee JJ, Tsimberidou AM, Hong DS, Naing A, Falchook GS, et al PIK3CA mutations frequently coexist with RAS and BRAF mutations in patients with advanced cancers. PLoS One. 2011; 6: e22769 10.1371/journal.pone.0022769 21829508 PMC3146490

[16] Eichhorn PJ, Gili M, Scaltriti M, Serra V, Guzman M, Nijkamp W, et al Phosphatidylinositol 3-kinase hyperactivation results in lapatinib resistance that is reversed by the mTOR/phosphatidylinositol 3-kinase inhibitor NVP-BEZ235. Cancer Res. 2008; 68: 9221–9230. 10.1158/0008-5472.CAN-08-1740 19010894 PMC2587064

[17] Sakr RA, Barbashina V, Morrogh M, Chandarlapaty S, Andrade VP, Arroyo CD, et al Protocol for PTEN expression by immunohistochemistry in formalin-fixed paraffin-embedded human breast carcinoma. Appl Immunohistochem Mol Morphol. 2010; 18: 371–374. 10.1097/PAI.0b013e3181d50bd5 20216404 PMC2921801

[18] Hernandez-Aya LF, Gonzalez-Angulo AM. Targeting the phosphatidylinositol 3-kinase signaling pathway in breast cancer. Oncologist. 2011; 16: 404–414. 10.1634/theoncologist.2010-0402 21406469 PMC3228119

[19] Maruyama N, Miyoshi Y, Taguchi T, Tamaki Y, Monden M, Arroyo CD, et al Clinicopathologic analysis of breast cancers with PIK3CA mutations in Japanese women. Clin Cancer Res. 2007; 13: 408–414. 17202311 10.1158/1078-0432.CCR-06-0267

[20] Perez-Tenorio G, Alkhori L, Olsson B, Waltersson MA, Nordenskjold B, Rutqvist LE, et al PIK3CA mutations and PTEN loss correlate with similar prognostic factors and are not mutually exclusive in breast cancer. Clin Cancer Res. 2007; 13: 3577–3584. 17575221 10.1158/1078-0432.CCR-06-1609

[21] Ellis MJ, Lin L, Crowder R, Tao Y, Hoog J, Davies S, et al Phosphatidyl-inositol-3-kinase alpha catalytic subunit mutation and response to neoadjuvant endocrine therapy for estrogen receptor positive breast cancer. Breast Cancer Res Treat. 2010; 119: 379–390. 10.1007/s10549-009-0575-y 19844788 PMC2810126

[22] Razis E, Bobos M, Kotoula V, Eleftheraki AG, Kalofonos HP, Pavlakis K, et al Evaluation of the association of PIK3CA mutations and PTEN loss with efficacy of trastuzumab therapy in metastatic breast cancer. Breast Cancer Res Treat. 2011; 128: 447–456. 10.1007/s10549-011-1572-5 21594665

[23] Jensen JD, Knoop A, Laenkholm AV, Grauslund M, Jensen MB, Santoni-Rugiu E, et al PIK3CA mutations, PTEN, and pHER2 expression and impact on outcome in HER2-positive early-stage breast cancer patients treated with adjuvant chemotherapy and trastuzumab. Ann Oncol. 2012; 23: 2034–2042. 10.1093/annonc/mdr546 22172323

[24] Baselga J, Cortes J, Kim SB, Im SA, Hegg R, Im YH, et al Pertuzumab plus trastuzumab plus docetaxel for metastatic breast cancer. N Engl J Med. 2012; 366: 109–119. 10.1056/NEJMoa1113216 22149875 PMC5705202

[25] Saal LH, Holm K, Maurer M, Memeo L, Su T, Wang X, et al PIK3CA mutations correlate with hormone receptors, node metastasis, and ERBB2, and are mutually exclusive with PTEN loss in human breast carcinoma. Cancer Res. 2005; 65: 2554–2559. 15805248 10.1158/0008-5472-CAN-04-3913

[26] Shoman N, Klassen S, McFadden A, Bickis MG, Torlakovic E, Chibbar R. Reduced PTEN expression predicts relapse in patients with breast carcinoma treated by tamoxifen. Mod Pathol. 2005; 18: 250–259. 15475931 10.1038/modpathol.3800296

[27] Depowski PL, Rosenthal SI, Ross JS. Loss of expression of the PTEN gene protein product is associated with poor outcome in breast cancer. Mod Pathol. 2001; 14: 672–676. 11454999 10.1038/modpathol.3880371

[28] Lee JS, Kim HS, Kim YB, Lee MC, Park CS, Min KW. Reduced PTEN expression is associated with poor outcome and angiogenesis in invasive ductal carcinoma of the breast. Appl Immunohistochem Mol Morphol. 2004; 12: 205–210. 15551732 10.1097/00129039-200409000-00004

[29] Iqbal J, Thike AA, Cheok PY, Tse GM, Tan PH. Insulin growth factor receptor-1 expression and loss of PTEN protein predict early recurrence in triple-negative breast cancer. Histopathology. 2012; 61:652–659. 10.1111/j.1365-2559.2012.04255.x 22759273

[30] Li SY, Rong M, Grieu F, Iacopetta B. PIK3CA mutations in breast cancer are associated with poor outcome. Breast Cancer Res Treat. 2006; 96: 91–95. 16317585 10.1007/s10549-005-9048-0

[31] Loi S, Haibe-Kains B, Majjaj S, Lallemand F, Durbecq V, Larsimont D, et al PIK3CA mutations associated with gene signature of low mTORC1 signaling and better outcomes in estrogen receptor-positive breast cancer. Proc Natl Acad Sci U S A. 2010; 107: 10208–10213. 10.1073/pnas.0907011107 20479250 PMC2890442

[32] Buzdar AU. Role of biologic therapy and chemotherapy in hormone receptor- and HER2-positive breast cancer. Ann Oncol. 2009; 20: 993–999. 10.1093/annonc/mdn739 19150946

[33] Osborne CK, Schiff R. Mechanisms of endocrine resistance in breast cancer. Annu Rev Med. 2011; 62: 233–247. 10.1146/annurev-med-070909-182917 20887199 PMC3656649

[34] Yamnik RL, Digilova A, Davis DC, Brodt ZN, Murphy CJ, Holz MK. S6 kinase 1 regulates estrogen receptor alpha in control of breast cancer cell proliferation. J Biol Chem. 2009; 284: 6361–6369. 10.1074/jbc.M807532200 19112174

[35] Crowder RJ, Phommaly C, Tao Y, Hoog J, Luo J, Perou CM, et al PIK3CA and PIK3CB inhibition produce synthetic lethality when combined with estrogen deprivation in estrogen receptor-positive breast cancer. Cancer Res. 2009; 69: 3955–3962. 10.1158/0008-5472.CAN-08-4450 19366795 PMC2811393

[36] Miller TW, Hennessy BT, Gonzalez-Angulo AM, Fox EM, Mills GB, Chen H, et al Hyperactivation of phosphatidylinositol-3 kinase promotes escape from hormone dependence in estrogen receptor-positive human breast cancer. J Clin Invest. 2010; 120: 2406–2413. 10.1172/JCI41680 20530877 PMC2898598

[37] Boulay A, Rudloff J, Ye J, Zumstein-Mecker S, O'Reilly T, Evans DB, et al Dual inhibition of mTOR and estrogen receptor signaling in vitro induces cell death in models of breast cancer. Clin Cancer Res. 2005; 11: 5319–5328. 16033851 10.1158/1078-0432.CCR-04-2402

[38] Baselga J, Campone M, Piccart M, Burris HA 3rd, Rugo HS, Sahmoud T, et al Everolimus in postmenopausal hormone-receptor-positive advanced breast cancer. N Engl J Med. 2012; 366: 520–529. 10.1056/NEJMoa1109653 22149876 PMC5705195

